# Novel AVPR2 mutations in congenital nephrogenic diabetes insipidus: clinical characteristics and genetic analysis

**DOI:** 10.3389/fped.2025.1623342

**Published:** 2025-09-08

**Authors:** Kunjiao Xue, Jin Wu, Juanjuan Lyu, Xiaomei Sun, Ying Liu, Chuanjie Yuan

**Affiliations:** ^1^ Department of Pediatrics, West China Second University Hospital, Sichuan University, Chengdu, China; ^2^ Key Laboratory of Birth Defects and Related Diseases of Women and Children (Sichuan University), Ministry of Education, Chengdu, China

**Keywords:** congenital nephrogenic diabetes insipidus, AVPR2, AQP2, novel mutation, treatment

## Abstract

**Objectives:**

Congenital nephrogenic diabetes insipidus (NDI) is a rare hereditary disorder caused by mutations in two critical genes: arginine vasopressin receptor 2 (AVPR2) and aquaporin 2 genes (AQP2). Mutations in AVPR2 gene, which are predominantly X-linked, account for a significant proportion of cases, particularly in men. Nevertheless, research on this condition in the western Chinese population remains limited.

**Methods:**

Eleven participants from nine families with NDI were screened for AVPR2 mutations. Their clinical features were documented, and genotype–phenotype associations were investigated.

**Results:**

This study included 11 pediatric patients with congenital NDI, comprising 10 boys and 1 girl. They were diagnosed between 1 month and 7 years of age. The clinical presentations included growth retardation, polydipsia, and polyuria in all patients (11), hypernatremia in 10, renal pelvis dilation in 4, absence of posterior pituitary high signal on magnetic resonance imaging in 3, unexplained fever in 3, and recurrent vomiting in 1 and mental retardation each in 1 patient. Genetic analysis revealed eight AVPR2 mutations among the 11 patients with congenital NDI, 3 of which were novel: p.Ile46Serfs*145, p.Ile177del, and p.Ser327Ilefs*30.

**Conclusions:**

In the largest case series of congenital NDI caused by AVPR2 mutations in the western Chinese population, eight AVPR2 mutations were identified, including three that were novel. This study enhances the existing literature by elucidating the clinical manifestations of congenital NDI by analyzing 11 cases and by identifying three novel mutation sites, thereby augmenting the genetic understanding of this condition.

## Introduction

Congenital nephrogenic diabetes insipidus (NDI) is caused by mutations in arginine vasopressin receptor 2 (AVPR2) and aquaporin 2 (AQP2) genes. The X-linked recessive inheritance pattern is observed in approximately 90% of cases with AVPR2 gene mutations, while AQP2 gene mutations, constituting approximately 10% of cases, exhibit either autosomal dominant or recessive inheritance. To date, more than 392 mutations in AVPR2 gene have been identified (1).

AVPR2 gene, located on the long arm of the X-chromosome (Xq28), spans 2.2 kb and comprises three exons. It encodes the type 2 arginine vasopressin receptor, a typical seven-transmembrane helical G protein-coupled receptor that mediates the antidiuretic effect of AVP (2). Children with congenital NDI often exhibit urinary concentration defects within the first few weeks after birth, presenting with polyuria and hypotonic urine. Unfortunately, these symptoms may not receive immediate attention, and most cases are diagnosed later due to growth impairment, recurrent vomiting, or recurrent fever (3). The clinical manifestations of congenital NDI often lack specificity, making the condition prone to oversight. If left untreated, complications may include urinary tract obstruction, dilation, intellectual impairment, and even death. Early diagnosis of this condition is crucial for effective intervention. This study provides a comprehensive summary of the clinical presentations and genetic characteristics of 11 congenital NDI cases observed at our institution, thereby significantly enhancing the understanding of this disorder.

## Materials and methods

### Subjects

This study summarizes 11 pediatric patients diagnosed with congenital NDI at the Department of Pediatric Genetic Metabolic Endocrinology, West China Second Hospital of Sichuan University, between December 2015 and December 2024. The diagnosis was established through a comprehensive evaluation that included family history, clinical manifestations, such as polyuria, polydipsia, hypernatremia, and low urine osmolality, results of water deprivation/desmopressin challenge tests, and genetic testing. Due to the unique clinical challenges in infants, such as the inability to cooperate with urine collection and difficulty in obtaining blood samples, we did not perform water deprivation/desmopressin challenge tests; instead, the diagnosis was mainly based on a comprehensive evaluation of clinical manifestations, genetic findings, and response to treatment.

### Genomic DNA sequencing and analysis

Whole-exome sequencing (WES) was performed on the proband and their family members. The identified variant sites were annotated using molecular biology techniques. Data were analyzed through the integration of pathogenic mutation databases, normal human genomic databases, clinical characteristics, and genetic data analysis algorithms. Finally, variants with clinical significance were selected for further analysis.

## Results

### Clinical manifestations of patients with congenital NDI

This study involved 11 children diagnosed with congenital NDI from nine families, including 10 boys and 1 girl. Demographic features and characteristics are reported in Table 1. The average age of onset was 1 year, with the main clinical manifestations being recurrent fever, polyuria, and growth retardation. Most patients had no apparent family history, except for two probands whose mother exhibited similar symptoms of polyuria.

**Table 1:** Clinical characteristics of patients with congenital NDI.

No.	Sex	Age (months)	Age at onset (months)	Polydipsia	Polyuria	Intermittent fever	Short stature	Mental impairment	Vomiting
1	Male	36	12	+	+	−	+	−	−
2	Male	94	36	+	+	−	+	−	−
3	Male	2	2	+	+	−	+	−	−
4	Male	10	7	+	+	+	+	−	−
5	Male	85	12	+	+	−	+	+	−
6	Male	32	6	+	+	−	+	−	−
7	Female	2	Neonatal period	+	+	+	+	−	−
8	Male	9	Neonatal period	+	+	−	+	−	+
9	Male	158	Neonatal period	+	+	+	+	−	−
10	Male	62	36	+	+	−	+	−	−
11	Male	4	Neonatal period	+	+	−	+	−	−

"+"means a patient with clinical manifestations; “−” means a patient without clinical manifestations.

### Auxiliary inspection characteristics of patients with congenital NDI

Biochemical and imaging characteristics of the patient cohort are presented in Table 2. All patients with congenital NDI exhibited decreased urine-specific gravity. Among them, 10 patients had blood sodium levels higher than normal (greater than 145 mmol/L), including 2 cases of mild hypernatremia (148.5 and 147.9 mmol/L), 2 cases of moderate hypernatremia (153 and 151.6 mmol/L), and 6 cases of severe hypernatremia (sodium level range: 157–173.6 mmol/L). One patient had a normal sodium level of 135 mmol/L. Urinary system ultrasound revealed renal hydronephrosis in three patients and renal collecting system dilation in one patient. All patients underwent pituitary magnetic resonance imaging (MRI), and three exhibited signs of absence of high signal intensity in the pituitary.

**Table 2:** Biochemical and radiological characteristics of patients with congenital NDI.

No.	Sex	Blood sodium (135–145 mmol/L)	Blood osmotic pressure (mOsm/kgH_2_O)	Urine-specific gravity (1.005–1.030)	Urine osmotic pressure (mOsm/kgH_2_O)	Uric acid (<320 μmol/L)	Urinary ultrasound	Pituitary MRI
1	Male	153.0	320.2	1.004	160	540	Normal	Normal
2	Male	157.0	327.6	1.002	80	533	Hydronephrosis	Rathke's sac
3	Male	165.2	336.0	1.002	80	268	Hydronephrosis	The high signal of the posterior pituitary disappears
4	Male	148.5	306.6	1.003	120	392	Normal	Normal
5	Male	173.6	359.8	1.002	80	221	Hydronephrosis	The high signal of the posterior pituitary disappears
6	Male	135.0	281.9	1.003	120	256	Normal	Normal
7	Female	162.5	322.5	1.002	80	297	Normal	Normal
8	Male	167.8	348.2	1.001	40	327	Renal collecting system dilation	Normal
9	Male	151.6	317.82	1.003	120	674	Normal	Normal
10	Male	147.9	309.72	1.004	160	457	Normal	Normal
11	Male	157.00	318.00	1.002	80	231	Normal	The high signal of the posterior pituitary disappears

### AVPR2 mutations in patients with congenital NDI

**Figure 1:**
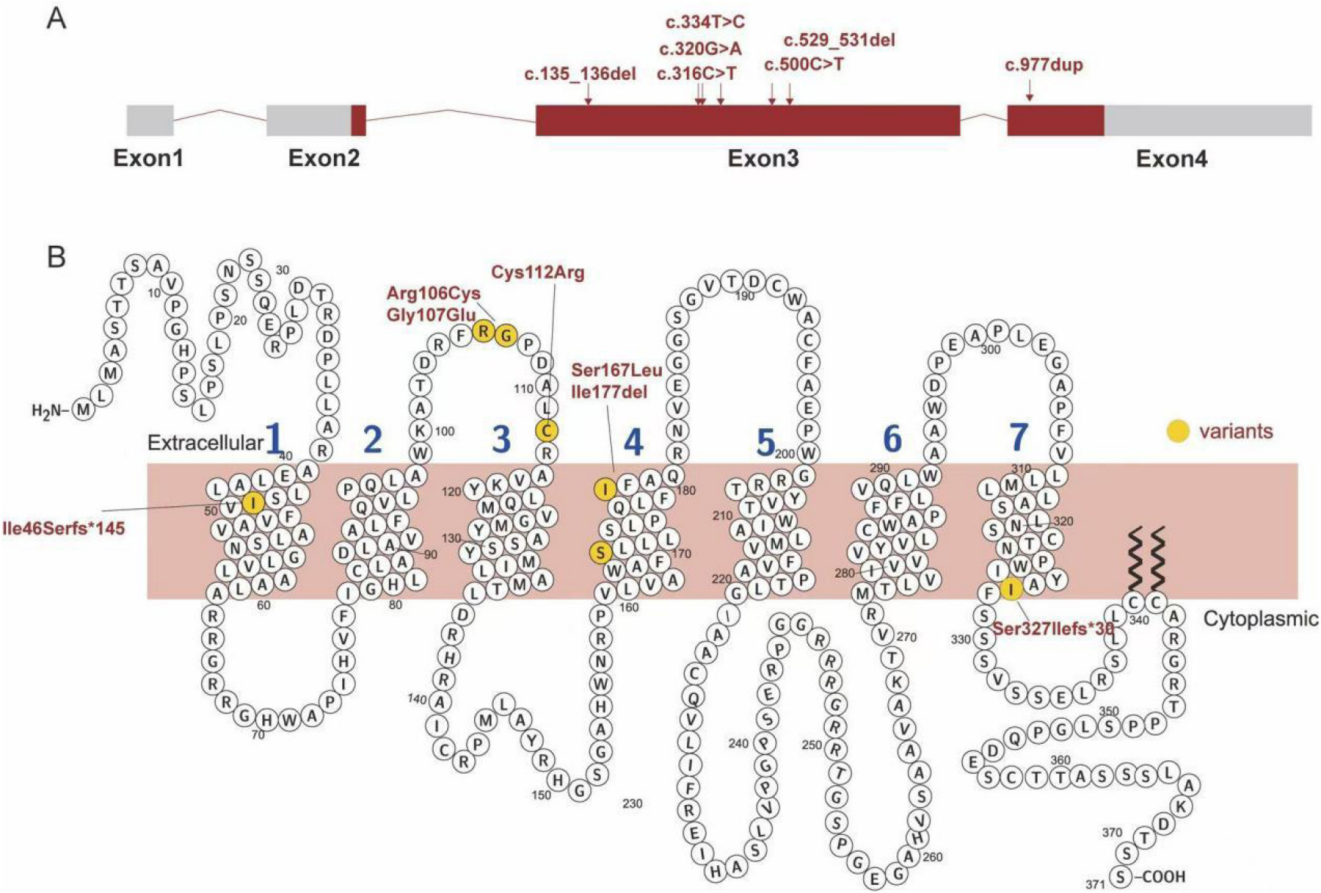
Distribution of AVPR2 gene mutations detected in NDI patients in this study. **(A)** Distribution of six mutations in AVPR2 gene. **(B)** Schematic of the primary structure of AVPR2 showing the location of the altered amino acids. Red rectangles indicate the DNA coding sequence, hollow circles indicate the amino acids of AVPR2, and yellow semicircles indicate the amino acid changes identified in the study.

**Figure 2:**
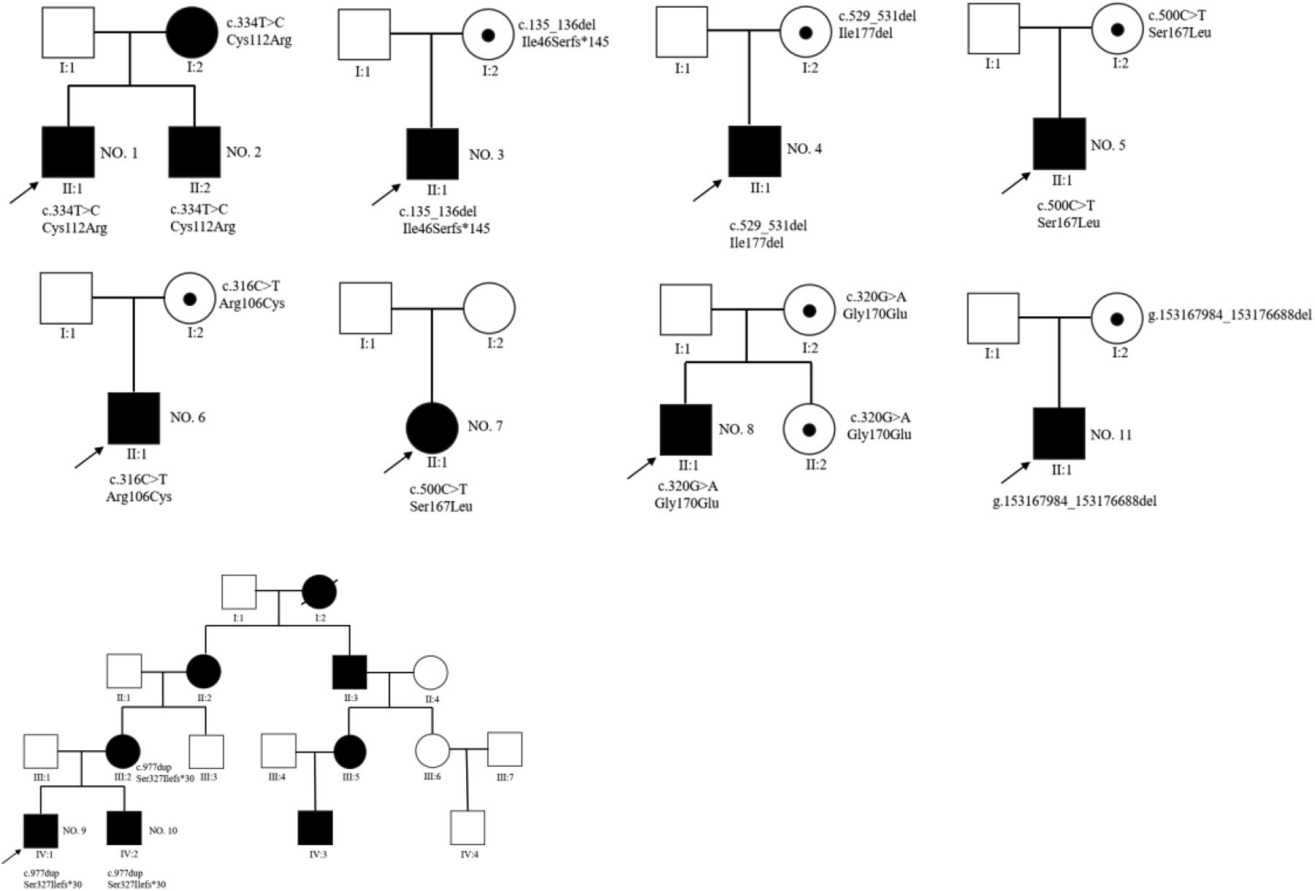
Pedigrees of the eight patients detected to carry pathogenic AVPR2 mutations.

Familial whole-exome sequencing was performed on 11 affected children from nine families, identifying 8 distinct AVPR2 mutations. Among these, missense mutations accounted for 54.5% (6/11), deletion mutations for 27.3% (3/11), and duplication mutations for 18.2% (2/11). The six missense mutations included two instances of c.500C>T (p.Ser167Leu), two of c.334T>C (p.Cys112Arg), one of c.316C>T (p.Arg106Cys), and one of c.320G>A (p.Gly170Glu). In addition, three new mutations were identified: a deletion mutation c.529_531del (p.Ile177del), a frameshift mutation c.135_136del (p.Ile46Serfs*145), and a duplication mutation c.977dup (p.Ser327Ilefs*30) (Table 3). The distribution of AVPR2 mutations is demonstrated in Figure 1. Only one patient harbored a *de novo* mutation, while the remaining inherited the mutated gene from heterozygous mothers. The pedigrees of these affected families with congenital NDI are displayed in Figure 2.

**Table 3:** Mutation analysis of AVPR2 in patients with congenital NDI.

No.	Sex	Type of mutation	Exon/intron no.	Nucleotide change	Amino acid change	ACMG	Inheritance	Family history	Reported
1	Male	Missense	Exon3	c.334T>C	Cys112Arg	VUS	Maternal	+	Yes (20, 21)
2	Male	Missense	Exon3	c.334T>C	Cys112Arg	VUS	Maternal	+	Yes (20, 21)
3	Male	Deletion	Exon3	c.135_136del	Ile46Serfs*145	P	Maternal	−	No
4	Male	Deletion	Exon3	c.529_531del	Ile177del	VUS	Maternal	−	No
5	Male	Missense	Exon3	c.500C>T	Ser167Leu	P	Maternal	−	Yes (22)
6	Male	Missense	Exon2	c.316C>T	106,Arg>Cys	LP	Maternal	−	Yes (23)
7	Female	Missense	Exon3	c.500C>T	Ser167Leu	P	*De novo*	−	Yes (24, 25)
8	Male	Missense	Exon3	c.320G>A	Gly170Glu	LP	Maternal	−	Yes (26)
9	Male	Duplication	Exon4	c.977dup	Ser327Ilefs*30	LP	Maternal	+	No
10	Male	Duplication	Exon4	c.977dup	Ser327Ilefs*30	LP	Maternal	+	No
11	Male	Deletion	—	g.153167984_153176688del	—	P	Maternal	—	Yes (27)

P, pathogenic; LP, likely pathogenic; VUS, variant of uncertain significance.

### Treatment

All patients were advised to adhere to a low-sodium diet and were educated regarding appropriate fluid intake and urinary habits. In addition, four patients were administered hydrochlorothiazide at a daily dosage of 3 mg/kg, while five patients received combination therapy with hydrochlorothiazide (3 mg/kg/day) and amiloride (0.3 mg/kg/day). In addition, two patients received indomethacin at a dose of 2 mg/kg/day. Urine output was successfully managed in all cases.

## Discussion

Congenital NDI is primarily caused by mutations in AVPR2 and AQP2 genes, with AVPR2 gene mutations accounting for the majority of cases (4). In this study, all 11 cases of congenital NDI resulted from AVPR2 gene mutations. AVPR2 gene, located on the q28 region of the X-chromosome, encodes the type 2 vasopressin receptor and comprises two exons and three introns. Approximately 392 AVPR2 mutations have been reported to cause congenital NDI (1). AVPR2 mutations can be classified into three types: Type 1 mutations involve receptors that reach the cell surface but exhibit impaired ligand binding and cannot induce normal cAMP production. Type 2 mutations involve receptors with intracellular transport defects, preventing them from reaching the cell surface and causing AVPR2 to be stored inside the cells. Type 3 mutations involve receptors that are inappropriately transcribed, leading to unstable mRNA that is quickly degraded (1, 5, 6). Pathogenic variations in AVPR2 gene primarily include missense mutations (approximately 55.8%), non-sense mutations (12.8%), and small frameshift deletions (10.4%) (5). This study identified six missense mutations in 11 cases, consistent with the predominant mutation types reported in the literature. In addition, three new mutations were detected: a deletion mutation c.529_531del (p.Ile177del), a frameshift mutation c.135_136del (p.Ile46Serfs*145), and a duplication mutation c.977dup (p.Ser327Ilefs*30). In children with similar clinical presentations, genetic testing facilitates early diagnosis and has important implications for clinical management and outcomes. However, due to current technical limitations, we have not yet conducted further functional studies of the identified mutations.

Congenital NDI caused by AVPR2 gene mutations follows an X-linked recessive inheritance pattern, resulting in a predominance of affected men (7). In this study, 10 out of the 11 patients were boys, and the mutations were inherited from their mothers. Of these, two lineages had a family history. Female patients carrying heterozygous AVPR2 gene mutations may exhibit varying degrees of congenital NDI-related symptoms due to X-chromosome inactivation bias (8). The study suggests that the combination of complete loss-of-function mutations and extremely skewed X inactivation is associated with severe X-linked congenital NDI. This reveals an association between clinical manifestations, mutation types, and X inactivation status (9). Accordingly, further determination of the degree of X-chromosome inactivation is valuable for understanding the clinical manifestations of female heterozygous patients.

Most patients with congenital NDI exhibit symptoms such as growth retardation, vomiting, polyuria, irritability, and intermittent fever accompanied by hypernatremic dehydration shortly after birth (10). Long-term effects may include intellectual disability and urinary tract obstruction with renal system dilation. In severe cases, hypernatremia can lead to death (11). Some studies indicate that the average onset age of congenital NDI is approximately 0–4 months (12). The onset age reported in this study ranged from a few days to 3 years after birth, with only 50% of patients diagnosed within the first year; this contrasts with existing literature, which emphasizes early diagnosis (usually within months) due to the severity of symptoms. The delayed diagnosis observed in some cases may be related to the delayed early recognition or the availability of genetic testing. Notably, all 11 patients demonstrated growth retardation. Some researchers believe that growth retardation may be related to the fact that children have a strong aversion to water, which limits the intake of high-calorie liquids or solids, resulting in weight gain and reduced linear growth (13). Three infants experienced unexplained fever during the neonatal period, and their body temperature gradually returned to normal after correction of hypernatremia, which was related to dehydration fever caused by hypernatremia. This observation aligns with the current reserch findings. Therefore, in infants presenting with unexplained fever during the neonatal period, electrolyte examination should be emphasized to avoid excessive investigations and treatments. In this study, only one child showed signs of intellectual disability, which was due to prolonged hypernatremia caused by delayed diagnosis. The incidence of urinary complications in children with congenital NDI is approximately 42%, with hydronephrosis and hydroureter being the most common findings (14). In this study, ultrasound examination revealed that 36% of patients had varying degrees of hydronephrosis, a slightly lower rate than previously reported. This difference may be attributed to the small sample size. Since the kidneys of children with congenital NDI excrete a large amount of urine, there is a greater possibility of urinary complications, particularly as blood sodium levels rise. The blood sodium levels of these four patients were significantly elevated. The mutations included p.Ile46Serfs*145, p.Ser167Leu, and p.Gly170Glu. It is speculated that patients with these mutations may have a more significant increase in blood sodium levels, which potentially increase the risk of urinary complications. Therefore, future studies are warranted to further explore the impact of mutations at these gene sites on AVPR2 protein function. On pituitary MRI, the posterior pituitary lobe appears to exhibit a high signal due to the presence of neurosecretory granules containing antidiuretic hormone. In patients with central diabetes insipidus, the high signal is absent, reflecting a lack or partial deficiency of antidiuretic hormone in the posterior pituitary lobe (15). In this study, none of the three patients exhibited high signals in the posterior pituitary on MRI, but the results of the water deprivation vasopressin test failed to confirm a diagnosis of central diabetes insipidus. Some studies have suggested that the disappearance of posterior pituitary hyperintensity may be related to the severity of the patient's condition (16). Compared with the other patients, the three patients with congenital NDI had relatively high plasma osmolality. We speculate that this increased plasma osmolality may have triggered the posterior pituitary to release vasopressin continuously, resulting in the disappearance of high signals on pituitary MRI, thus producing imaging results similar to those seen in central diabetes insipidus. Unfortunately, the current data cannot further confirm this hypothesis. We hope to collect further follow-up data in the next phase to observe changes in cranial imaging after treatment in these children.

Currently, there is no definitive treatment for congenital NDI; thus, treatment primarily focuses on symptomatic management to improve overall symptoms (17). In addition to adopting a low-salt and low-protein diet, it is especially important to ensure adequate caloric and protein intake to promote normal growth and development in children with congenital NDI (12). Preventing dehydration and avoiding hypernatremia are crucial aspects of care. Thiazide diuretics function by inhibiting the thiazide-sensitive cotransporter in the distal tubule, thereby reducing salt reabsorption. As sodium loss decreases plasma volume, less water enters the collecting ducts, ultimately reducing urine output (18, 19). In this study, four patients were administered hydrochlorothiazide monotherapy, while five received combination therapy with hydrochlorothiazide and amiloride. The remaining two patients, who presented with hyperuricemia, were treated with indomethacin. All patients exhibited significant amelioration of polydipsia and polyuria symptoms following treatment, with their blood sodium levels, as monitored, returning to normal ranges. In addition, none of the patients experienced hypokalemia during the course of treatment. New treatments for congenital NDI remain in the research and exploration stage, and the clinical data are relatively small. Researchers may explore gene therapy, cell therapy, or other new drug therapies, but these methods require more experimental research and clinical trials to verify their safety and efficacy.

This study included only 11 patients, representing a small sample size. All enrolled children showed growth retardation, and further follow-up is needed to assess their growth trajectories. In addition, considering the huge geographical and socioeconomic differences in the western region (such as urban–rural differences), future research may explore whether these factors affect the severity of the clinical phenotype of NDI.

## Conclusion

In summary, we performed a mutation analysis in a cohort of patients with congenital NDI from western China and identified eight AVPR2 gene mutations across nine families, including three novel mutations. The clinical, biochemical, and imaging characteristics of these patients were examined, revealing a correlation between genotype and phenotype. These findings expand the known genotype–phenotype spectrum of rare NDI caused by AVPR2 mutations and underscore the need for further research into the molecular biology of AVPR2.

## Data Availability

The data presented in the study are deposited in the ClinVar repository, accession numbers SCV006310933-SCV006310939.
